# Quantitative and Chemical Fingerprint Analysis for the Quality Evaluation of Platycodi Radix Collected from Various Regions in China by HPLC Coupled with Chemometrics

**DOI:** 10.3390/molecules23071823

**Published:** 2018-07-23

**Authors:** Haiyang Lu, Mengzhen Ju, Shanshan Chu, Tao Xu, Yuzhe Huang, Qingyun Chan, Huasheng Peng, Shuangying Gui

**Affiliations:** 1 College of Pharmacy, Anhui University of Chinese Medicine, Hefei 230012, China; lhy668866@163.com (H.L.); jmz_ahtcm@163.com (M.J.); cshan0916@126.com (S.C.); xutaojy@126.com (T.X.); pfhuangyz@163.com (Y.H.); chanqingyun126@126.com (Q.C.); 2 Synergetic Innovation Center of Anhui Authentic Chinese Medicine Quality Improvement, Hefei 230038, China; 3 Institute of Pharmaceutics, Anhui Academy of Chinese Medicine, Hefei 230012, China

**Keywords:** Platycodi Radix, quality evaluation, quantitative analysis, chemical fingerprint analysis, saponin, high performance liquid chromatography (HPLC), chemometrics

## Abstract

Platycodi Radix (PR) is the root of *Platycodon grandiflorum* (Jacq.) A. DC., which has been used for a long time in China to treat pulmonary diseases. The present study aimed to evaluate the quality of PR samples collected from 23 regions of 11 provinces in China. Eight saponins were quantified using HPLC coupled with evaporative light scattering detection (HPLC-ELSD). The samples with the highest total contents of saponins were from southern China, such as Yunnan, Guangxi, Jiangxi, and Guangzhou. The fingerprint analysis of PR samples was conducted by HPLC-UV method. Nineteen common peaks were selected and the similarity values varied from 0.607 to 0.921. These findings indicated that the saponins contents of PR from different regions varied significantly, with PR samples from southern China having the highest contents of saponins. These comprehensive methods were successful in evaluating the quality of PR samples from northern and southern China, which will serve as a guide for the development of PR as a clinical medication.

## 1. Introduction

Platycodi Radix (PR) is the root of *Platycodon grandiflorum* (Jacq.) A.DC., which has been used for a long time in China to treat pulmonary diseases. PR is increasingly being used not only as a traditional herbal medicine, but also as a popular functional food due to its rich content of amino acids. It is often made into pickles in Northeast China, Japan, Korea, and other East Asian countries. According to several studies, the starch of PR can be made into high-quality pastry and PR extracts can be used as active ingredients in anti-aging cosmetics and body lotion [1,2,3]. PR is distributed all over China, from the south to the north. In Chinese books of herbal medicine, PR from eastern China was called “Nan jiegeng” and presumed of better quality, while that distributed in north China and northeast China was called “Bei jiegeng” with larger production. In addition, PR from the south has bitter taste and good quality, according to the ancient book of herbal medicine. PR is an edible plant which can be used as both medicine and food. PR with bitter taste have been usually used as medicine, and that with sweet taste have been usually used as food [4]. Chemical compounds that have been isolated from PR include triterpenoid saponins, polysaccharide, flavonoids, phenolic compounds, inorganic elements, steroids, fatty acid, volatile oil, and other bioactive compounds [5,6,7,8,9]. Approximately 20 kinds of platycosides, the main bioactive constituents of PR, are responsible for a variety of biological activities such as anti-inflammation, antitumor, anti-allergy, anti-obesity, antioxidant, and antihyperlipidemic activities [10,11,12,13].

Several studies have reported that herbs collected at different times of year, or planted in different geographical environments might differ in quantity of chemical components and quality of medicinal materials [14,15]. Therefore, a comprehensive analysis on the quality control of PR is necessary. High Performance Liquid Chromatography coupled with evaporative light scattering detection (HPLC-ELSD) can detect most compounds regardless of their optical properties, which is effective for simultaneous determination of saponin contents [16,17]. According to Chinese Pharmacopeia (2015), the content of platycoside D in PR should not be less than 0.1%. However, several marker compounds are difficult to evaluate a sample without any information about the other components [18,19]. HPLC fingerprint analysis has been reported to assess the quality of the whole compositions of PR. Chromatographic fingerprint analysis is a useful method for the identification and quality control of botanical medicines [20,21].

The present study aimed to: (1) analyze the variation in saponin profiles of PR collected from 23 regions and 11 provinces in China using hierarchical clustering analysis (HCA) and principal component analysis (PCA) methods; (2) establish an effective HPLC-UV method for the identification and quality evaluation of PR based on HPLC fingerprint analysis; and (3) provide a theoretical basis to classify PR from different regions based on its medicinal quality by chemometrics method.

## 2. Results and Discussion

### 2.1. Optimization of Extraction

The optimization of extraction procedure included the optimization of solvents water, ethanol, methanol, 70% methanol (methanol/water 70:30, *v*/*v*) and extraction times (1, 2 and 3 times). The total contents of eight types of saponins was used as a marker for the evaluation of extraction efficiency. Table 1 shows that 70% MeOH displayed the highest extraction efficiency, whereas EtOH displayed the lowest. The total contents of saponins increased as the ultrasound duration increased (Table 2).

### 2.2. Method Validation

A developed HPLC-ELSD method was used to assess the repeatability, precision, recovery, stability, and linear ranges of method to determine contents of eight saponins. The results indicated a positive correlation between the natural logarithm of the investigated compounds’ mass and that of their peak areas within the test ranges (R^2^ > 0.9974) (Table 3). The precision (RSD) value of the method was less than 2.84%. The stability and repeatability (RSD) values were both less than 2.94%. The method recovery (RSD) value was less than 2.59% (Table 4). The results showed that the method was precise and accurate for the quantitative determination of eight saponins contents in PR from different regions.

### 2.3. Quality Evaluation of PR in China

#### 2.3.1. Quantification of Eight Investigated Compounds in PR

**Figure 1:**
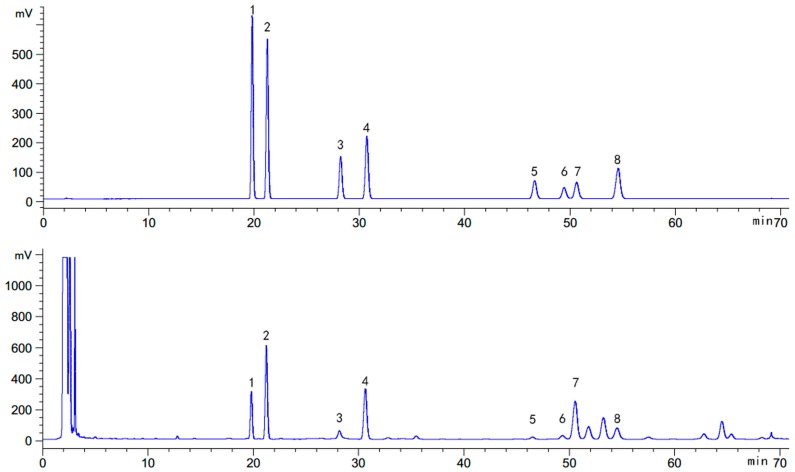
Peaks identification: (**1**) Deapioplatycoside E; (**2**) Platycoside E; (**3**) Deapioplatycodin D_3_; (**4**) Platycodin D_3_; (**5**) Deapioplatycodin D; (**6**) Platycodin D_2_; (**7**) Platycodin D; (**8**) Polygalacin D.

The developed HPLC-ELSD method was applied for the quantitative determination of 8 types of saponins in 89 PR samples. The saponins in PR samples were well separated using the developed HPLC method based on the comparison of their retention time with the reference substances. Peaks **1**, **2**, **3**, **4**, **5**, **6**, **7**, and **8** were identified as deapioplatycoside E, platycoside E, deapioplatycodin D_3_, platycodin D_3_, deapioplatycodin D, platycodin D_2_, platycodin D, and polygalacin D, respectively (Figure 1). The contents of the eight types of saponins in PR samples collected from 23 regions in China are summarized in Supplementary Table S1.

As shown in Supplementary Table S1, there was a large difference in the contents of the eight saponins from all samples. Deapioplatycoside E contents ranged from 0.092 to 2.772 mg/g, whereas platycoside E contents were within a range of 0.335–4.291 mg/g. Deapioplatycodin D_3_ contents in most samples were lower than 1 mg/g. Platycodin D_3_ contents were within a range of 0.282–5.650 mg/g. Deapioplatycodin D and platycodin D_2_ contents were lower than 1.623 mg/g and 1.355 mg/g, respectively. The contents of platycodin D ranged from 0.234 to 6.822 mg/g, whereas polygalacin D contents were within a range of 0.195–2.171 mg/g. Thus, the compounds with higher concentration in the samples were deapioplatycoside E, platycoside E, platycodin D_3_, platycodin D, and polygalacin D.

**Figure 2:**
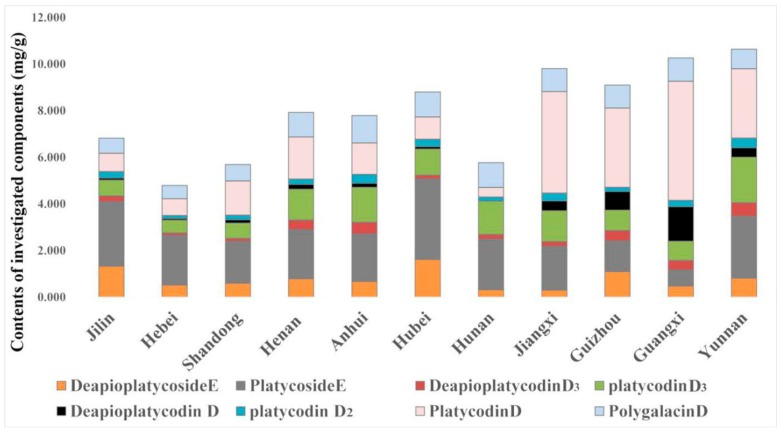
Comparison of eight investigated compounds in samples from 11 provinces.

Due to individual differences of each sample, it was difficult to exactly determine the quality of PR from the 11 provinces. To evaluate the quality of samples, the average contents of all were selected (Table 5, Figure 2).

The results showed that saponins contents in PR from the different regions greatly varied, as shown in Table 5 and Figure 2. According to Chinese Pharmacopeia (2015), the content of platycoside D in PR should not be less than 0.1%. The content of platycodon D in samples from four provinces (Jilin, Hebei, Hubei, Hunan) did not reach this criterion. The samples with the lowest total contents of the eight saponins were from Hebei (4.792 mg/g), followed by samples from Shandong (5.692 mg/g), Hunan (5.778 mg/g), and Jilin (6.824 mg/g), whereas the samples with the highest total contents of the eight saponins were from Yunnan (10.647 mg/g), followed by samples from Guangxi (10.269 mg/g), Jiangxi (9.816 mg/g), and Guangzhou (9.106 mg/g). The four PR samples with higher contents of saponin were considered of higher quality. The most abundant types of saponins in the PR samples, namely deapioplatycoside E, platycoside E, platycodin D_3_, platycodin D, and polygalacin D, could be the main ingredients of PR and might be suitable for quality evaluation of PR.

#### 2.3.2. Hierarchical Clustering Analysis (HCA)

**Figure 3:**
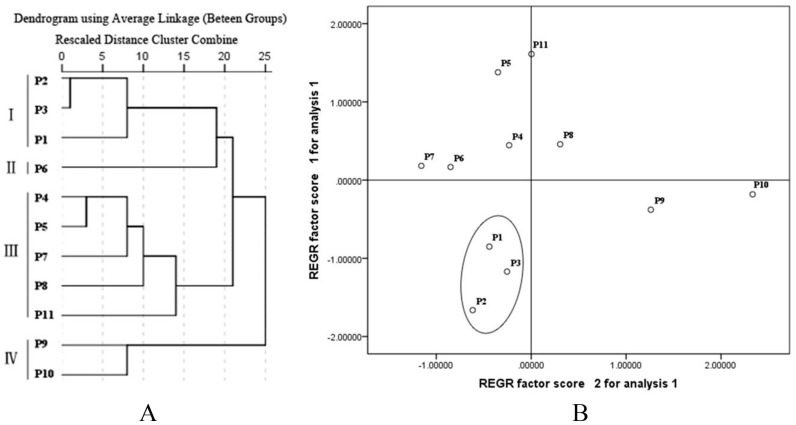
Results of hierarchical cluster analysis and principal component analysis of the samples from 11 provinces. (**A**: hierarchical cluster analysis; **B**: principal component analysis).

HCA of the samples from 11 provinces was performed based on their eight saponins contents. Figure 3 clearly shows that the 11 provinces could be divided into four clusters: Hebei, Shandong, and Jilin, with the lowest total contents of eight saponins, were classified into one cluster (Cluster I). The geographical locations of these provinces reflected the low quality of the samples from these regions. Jiangxi, Guizhou, Guangxi, and Yunnan, with the highest total contents of eight saponins, were classified into cluster III and IV, indicating the high quality of the samples from these provinces.

#### 2.3.3. Principal Component Analysis (PCA)

In order to evaluate the homogeneity of the quality of PR samples from the different regions of China, PCA was performed. The scatter points (Figure 3) showed that the results were consistent with that of HCA, in which the samples from Jilin, Hebei, and Shandong were distributed together, suggesting the homogeneity of PR quality from these regions, which was low due to their low contents of saponins. Obviously, these provinces could be distinguished from the other regions through the scatter diagram. Therefore, the eight saponins contents of PR have a potential use as markers for the quality assessment of PR.

### 2.4. Establishment of Chromatographic Fingerprint of PR and Similarity Analysis (SA)

**Figure 4:**
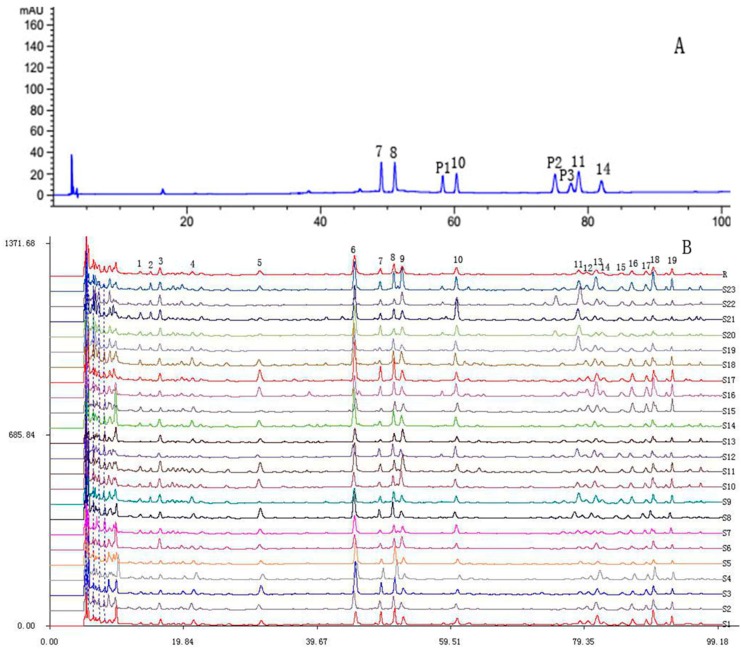
(**A**): 7 Deapioplatycoside E; 8 Platycoside E; P1 Deapioplatycodin D_3_; 10 Platycodin D_3_; P2 Deapioplatycodin D; P3 Platycodin D_2_; 11 Platycodin D; 14 Polygalacin D; (**B**): The chromatographic fingerprint of PR from 23 locations.

To standardize the HPLC profile, the samples of each origin were analyzed, and all chromatograms were referred to the “Similarity Evaluation System for Chromatographic Fingerprint of Traditional Chinese Medicine” (Version 2004A, Chinese Pharmacopoeia Commission, Beijing, China). The chromatographic fingerprint of PR from 23 locations was shown in Figure 4B, in which there were 19 distinct common peaks with clear separations from the common patterns in the 23 samples of each origin. Five common peaks (peak 7, 8, 10, 11, and 14) were identified as deapioplatycoside E, platycoside E, platycodin D_3_, platycodin D, and polygalacin D, respectively by comparing their retention time with the reference substances (Figure 4A). Low contents of deapioplatycodin D_3_ (peak P1), deapioplatycodin D (peak P2), and platycodin D_2_ (peak P3) were not shown in the chromatographic fingerprint.

The comparison between the standard fingerprint of the different samples showed similarity values ranged from 0.607 to 0.921 (Table 6). The samples S3, S5, and S15 have similarity values of less than 0.631, whereas the other samples have similarity values of more than 0.749. The results indicated the composition and content of chemical compounds in the PR of 23 locations varied significantly.

## 3. Materials and Methods

### 3.1. Samples and Reagents

**Figure 5:**
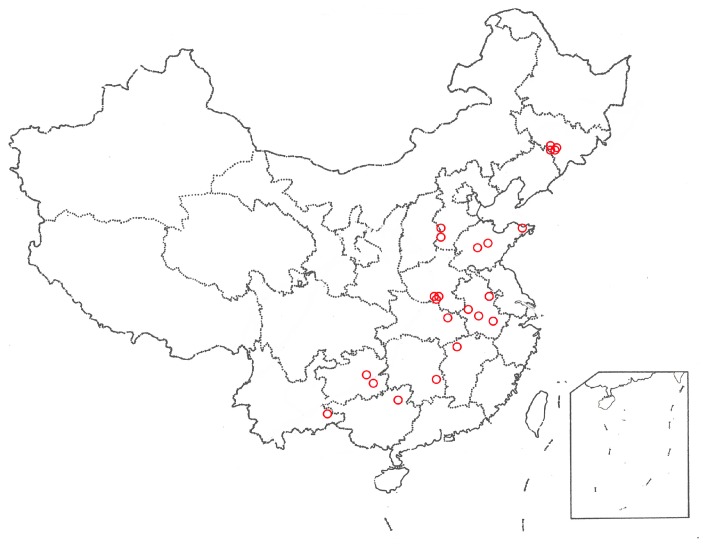
PR sampling locations in different regions in China.

**Figure 6:**
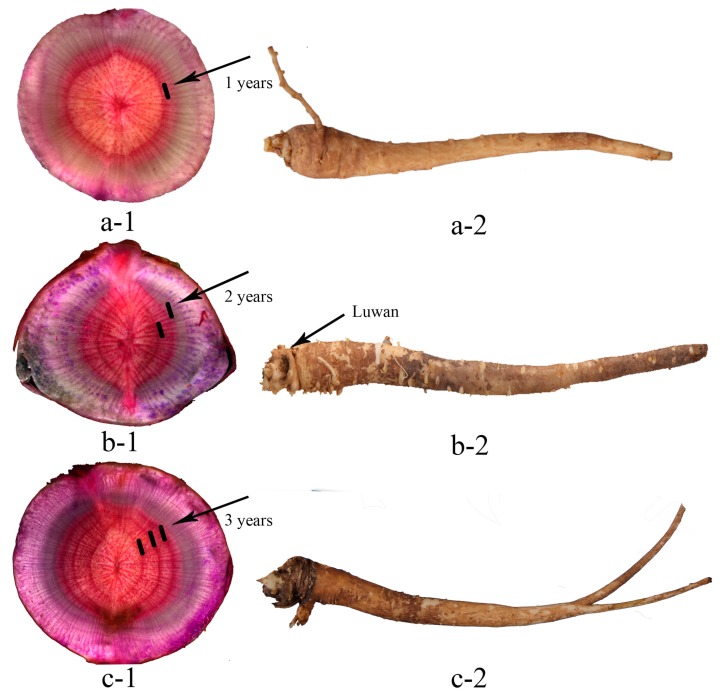
The determination of growth years of PR samples (1. transverse section of root; 2. root; a. one-year root; b. two-years root; c. three-years root.).

Wild PR samples were collected from 23 regions in 11 different provinces in China during September–October (Table 7, Figure 5). The growth years of all samples were determined by the bowl-shaped stem marks (known as “Luwan” in Chinese), and some samples showed clear growth rings after phloroglucinol staining method [22] (Figure 6). PR samples were authenticated as the root of *Platycodon grandiflorum* by Professor Huasheng Peng and deposited to Synergetic Innovation Center of Anhui Authentic Chinese Medicine Quality Improvement, Anhui University of Chinese Medicine, Hefei, China.

HPLC-grade acetonitrile was purchased from Sinopharm Chemical Reagent Co., Ltd. (Shanghai, China). HPLC-grade water was prepared using a Milli-Q water purification system from Pall Filter Co., Ltd. (Beijing, China). All other reagents were of analytical grade. Platycoside E, deapioplatycoside E, platycodin D_3_, and deapioplatycodin D_3_ were purchased from Chengdu Push Biotechnology Co., Ltd. (Chengdu, China). Platycodin D was purchased from Chengdu Must Bio-Technology Co., Ltd. (Chengdu, China). Deapioplatycodin D, platycodin D_2_, and polygalacin D were purchased from Chengdu Biopurify Phytochemicals, Ltd. (Chengdu, China). The purity of the all saponins was higher than 98%.

### 3.2. Sample Preparation

PR samples were vacuum-dried in an oven (Type DZF-6050, Shanghai Boxun Industry and Commerce Co., Ltd., Shanghai, China) at 50 °C and were ground into fine powder (50 mesh) using a powdering machine (Type RHP-250A, Zhejiang Yongkang Ronghao Industy Trade Co., Ltd., Yongkang, China). Subsequently, different extraction solvents (methanol, 70% methanol, ethanol, water) and extraction times (1, 2, and 3 times) were evaluated for the optimization of PR extract (Table 1 and Table 2). PR power (1.5 g) was extracted thrice with 30 mL of 70% methanol by ultrasonic at 25 °C for 30 min. The extract was filtered and evaporated. The resulting residue was dissolved in 10 mL 50% methanol (methanol/water 50:50, *v*/*v*) solution, and then filtered through a 0.45 μm Nylon filter prior to HPLC analysis with an injection volume of 30 μL.

### 3.3. HPLC-ELSD Instrumentation and Chromatographic Conditions

All analyses were performed on an Agilent Series 1260 system (Agilent Technologies, Santa Clara, CA, USA), equipped with a vacuum degasser, quaternary pump, autosampler, column compartment, and evaporative light-scattering detector, controlled by Agilent 1260 LC Software (OpenLab chemstation C. 01. 07, Santa Clara, CA, USA). A Zorbax Eclipse XDB-C18 column (250 mm × 4.6 mm, 5 μm particle size) was used for chromatography at 30 °C. Gradient elution was conducted with (A) water and (B) acetonitrile as mobile phases. The gradient was as follows: 0–15 min, 15–21% B; 15–37 min, 21–23% B; 37–52 min, 23–24% B; 52–65 min, 24–26% B; 65–70 min, 26–100% B; 70–75 min, 100–15% B; 75–85 min, 15–15% B. The flow rate was 1.0 mL/min, the sample injection volume was 30 μL, and the column temperature was 35 °C. HPLC-ELSD was to detection of 8 saponins with a gas spray nebulizer temperature of 50 °C, drift tube temperature of 85 °C, N_2_ gas pressure of 55 psi.

### 3.4. Method Validation of the Quantitative Analysis

Six replicates of the same sample were collected for the repeatability validation of the processing method of the sample solution. The method’s precision was validated by injecting the same sample solution six times. The stability of samples solutions was analyzed at 1, 2, 4, 8, 12, and 24 h after their preparation. To determine the recovery of the method, eight mixed standard solutions of saponin were added to the six samples by the same method as the sample-solution processing. The standards (deapioplatycoside E 4.30 mg, platycoside E 5.00 mg, deapioplatycodin D_3_ 2.30 mg, platycodin D_3_ 3.77 mg, deapioplatycodin D 1.78 mg, platycodin D_2_ 2.10 mg, platycodin D 8.00 mg, polygalacin D 3.65 mg) were dissolved in 10 mL 50% methanol and diluted to 1, 2, 4, 6, 8, and 10 times concentration to establish the standard curve for calibration. The sample injection volume was 30 μL.

### 3.5. Fingerprint Analysis

The fingerprint analysis of all samples was performed by HPLC-UV method. Equal amount of powder (1.0 g) from each origin was used as the sample for fingerprint analysis. Gradient elution system was conducted with (A) 0.1% phosphoric acid and (B) acetonitrile as mobile phases. The gradients were as follows: 0–23 min, 15–15% B; 23–45 min, 15–21% B; 45–55 min, 21–23% B; 55–80 min, 23–25% B; 80–100 min, and 25–35% B. The flow rate was 0.8 mL/min, the sample injection volume was 20 μL, the column temperature was 30 °C, and the detection wavelength was 210 nm [23].

### 3.6. Data Analysis

HCA and PCA were performed by using SPSS 20.0 (SPSS Inc., Chicago, IL, USA). The 8 saponins contents of PR samples from 11 provinces were determined in order to differentiate and classify PR samples of various origins. These data were standardized before HCA and PCA. First and second principal components were selected to draw a scatter plot for PCA. The average linkage between groups method was applied for HCA, with square euclidean distance used to measure the distance matrix between observations [24].

Nineteen characteristic peaks in the chromatograms were selected. The reference fingerprint and similarity among tested samples were calculated and generated by a Similarity Evaluation System for Chromatographic Fingerprint of Traditional Chinese Medicine (Version 2004A, Chinese Pharmacopoeia Commission, Beijing, China) software, which was recommended by the State Pharmacopoeia Commission of the People’s Republic of China [25].

## 4. Conclusions

In this paper, a rapid and reliable HPLC-ELSD method was developed for the simultaneous determination of 8 saponins contents and chromatographic fingerprint analysis was performed to evaluate the quality of PR samples from various origins in China. The samples with the highest saponins contents were from southern China. All samples in the 11 provinces of China were classified into four groups based on their contents of 8 saponins using hierarchical clustering method. The resulting classification was closely associated to the geographical environment of the regions.

## Figures and Tables

**Table 1:** The investigation of different extraction solvents.

Solvent Systems	The Contents of Each Compound ^a^ (mg/g)							
	1	2	3	4	5	6	7	8
Water	0.949	1.924	0.481	1.225	0.698	0.165	3.506	0.963
MeOH	0.931	1.846	0.534	1.596	0.564	0.178	3.501	1.675
70% MeOH	1.501	2.882	0.860	2.525	0.936	0.233	5.390	2.017
EtOH	0.181	0.396	0.192	0.413	0.232	- ^b^	1.546	0.927

^a^ (**1**) Deapioplatycoside E; (**2**) Platycoside E; (**3**) Deapioplatycodin D_3_; (**4**) Platycodin D_3_; (**5**) Deapioplatycodin D; (**6**) Platycodin D_2_; (**7**) Platycodin D; (**8**) Polygalacin D; ^b^ undetected.

**Table 2:** The investigation of different extraction times.

Extraction Times	The Content of Each Compound ^a^ (mg/g)							
	1	2	3	4	5	6	7	8
One	1.342	2.629	0.614	2.016	0.830	- ^b^	4.852	1.755
Two	1.655	3.170	0.963	2.545	1.061	0.314	6.079	2.165
Three	1.739	3.355	1.043	2.739	1.062	0.262	6.264	2.189

^a^ (**1**) Deapioplatycoside E; (**2**) Platycoside E; (**3**) Deapioplatycodin D_3_; (**4**) Platycodin D_3_; (**5**) Deapioplatycodin D; (**6**) Platycodin D_2_; (**7**) Platycodin D; (**8**) Polygalacin D. ^b^ undetected.

**Table 3:** The results of the regression equation, R^2^ and linearity range of eight saponins.

Compound ^a^	Regression Equation	R^2^	Linearity Range( μg)
**1**	y = 1.4275x + 6.0015	0.9974	1.290~12.900
**2**	y = 1.448 x + 5.7217	0.9977	1.500~15.000
**3**	y = 1.5908x + 5.6015	0.9978	0.690~6.900
**4**	y = 1.556 x + 5.3293	0.9975	1.131~11.310
**5**	y = 1.6544x + 5.3848	0.9982	0.534~5.340
**6**	y = 1.6606x + 5.1075	0.9976	0.630~6.300
**7**	y = 1.464x + 5.0489	0.9999	2.400~24.000
**8**	y = 1.6435x + 4.9158	0.9989	1.095~10.950

^a^ (**1**) Deapioplatycoside E; (**2**) Platycoside E; (**3**) Deapioplatycodin D_3_; (**4**) Platycodin D_3_; (**5**) Deapioplatycodin D; (**6**) Platycodin D_2_; (**7**) Platycodin D; (**8**) Polygalacin D.

**Table 4:** The results of the precision, stability, repeatability, and recovery tests.

Compound ^a^	Precision (RSD, %)	Stability (RSD, %)	Repeatability (RSD, %)	Recovery (RSD, %)
**1**	0.65	2.73	1.07	1.89
**2**	1.00	2.85	1.65	1.52
**3**	2.55	0.97	1.22	1.86
**4**	0.72	1.71	2.94	1.93
**5**	2.79	1.82	1.48	2.13
**6**	1.80	2.48	2.70	2.59
**7**	1.57	1.02	1.21	1.54
**8**	2.84	2.94	1.84	1.37

^a^ (**1**) Deapioplatycoside E; (**2**) Platycoside E; (**3**) Deapioplatycodin D_3_; (**4**) Platycodin D_3_; (**5**) Deapioplatycodin D; (**6**) Platycodin D_2_; (**7**) Platycodin D; (**8**) Polygalacin D. RSD = relative standard deviation.

**Table 5:** Contents (mg/g) of eight investigated compounds in samples from 11 provinces.

Code	Province	The Average Contents of each Compound ^a^ (mg/g)								Total
		1	2	3	4	5	6	7	8	
P1	Jilin	1.324 ± 0.735	2.774 ± 0.707	0.247 ± 0.135	0.682 ± 0.299	0.081 ± 0.091	0.278 ± 0.149	0.787 ± 0.494	0.651 ± 0.227	6.824
P2	Hebei	0.516 ± 0.228	2.133 ± 0.963	0.103 ± 0.070	0.559 ± 0.161	0.064 ± 0.055	0.129 ± 0.083	0.717 ± 0.307	0.571 ± 0.629	4.792
P3	Shandong	0.593 ± 0.440	1.796 ± 0.362	0.134 ± 0.109	0.656 ± 0.261	0.137 ± 0.161	0.198 ± 0.149	1.469 ± 1.011	0.709 ± 0.245	5.692
P4	Henan	0.788 ± 0.469	2.103 ± 0.786	0.419 ± 0.272	1.323 ± 0.570	0.200 ± 0.235	0.232 ± 0.149	1.807 ± 1.293	1.060 ± 0.472	7.932
P5	Anhui	0.669 ± 0.568	2.040 ± 0.985	0.505 ± 0.854	1.501 ± 1.264	0.169 ± 0.314	0.381 ± 0.362	1.351 ± 1.101	1.180 ± 0.610	7.796
P6	Hubei	1.616 ± 0.890	3.462 ± 0.977	0.156 ± 0.016	1.122 ± 0.330	0.103 ± 0.035	0.318 ± 0.082	0.954 ± 0.277	1.080 ± 0.078	8.811
P7	Hunan	0.313 ± 0.022	2.172 ± 0.553	0.209 ± 0.009	1.428 ± 0.638	- ^b^	0.177 ± 0.008	0.409 ± 0.110	1.070 ± 0.342	5.778
P8	Jiangxi	0.296 ± 0.083	1.883 ± 0.512	0.207 ± 0.009	1.323 ± 0.525	0.418 ± 0.182	0.335 ± 0.058	4.362 ± 1.667	0.992 ± 0.353	9.816
P9	Guizhou	1.096 ± 0.763	1.321 ± 0.713	0.439 ± 0.269	0.880 ± 0.324	0.795 ± 0.381	0.185 ± 0.044	3.402 ± 0.973	0.988 ± 0.248	9.106
P10	Guangxi	0.473 ± 0.338	0.691 ± 0.374	0.413 ± 0.083	0.827 ± 0.242	1.477 ± 0.146	0.269 ± 0.088	5.113 ± 0.905	1.006 ± 0.213	10.269
P11	Yunnan	0.810 ± 0.411	2.656 ± 1.172	0.592 ± 0.261	1.951 ± 0.489	0.399 ± 0.100	0.418 ± 0.056	2.978 ± 0.751	0.843 ± 0.057	10.647

^a^ (**1**) Deapioplatycoside E; (**2**) Platycoside E; (**3**) Deapioplatycodin D_3_; (**4**) Platycodin D_3_; (**5**) Deapioplatycodin D; (**6**) Platycodin D_2_; (**7**) Platycodin D; (**8**) Polygalacin D. ^b^ undetected.

**Table 6:** The similarities of the chromatograms of PR from each location.

Samples	Similarities	Samples	Similarities
S1	0.873	S13	0.900
S2	0.775	S14	0.888
**S3**	**0.631**	**S15**	**0.607**
S4	0.769	S16	0.876
S5	0.610	S17	0.879
S6	0.879	S18	0.887
S7	0.895	S19	0.875
S8	0.916	S20	0.902
S9	0.793	S21	0.872
S10	0.837	S22	0.749
S11	0.919	S23	0.921
S12	0.838		

**Table 7:** PR samples collected from different regions of China.

NO.	Origin	Coordinates	Number of samples	Code
S1	Lalahe, Dongfeng, Jilin	N 42°39′ E 125°23′	5	JL1-1~JL1-5
S2	Hengdaohe, Dongfeng, Jilin	N 42°24′ E 125°17′	6	JL2-1~JL2-6
S3	Xiaoyang, Meihekou, Jilin	N 42°18′ E 125°24′	4	JL3-1~JL3-4
S4	Shuidao, Meihekou, Jilin	N 42°18′ E 125°32′	4	JL4-1~JL4-4
S5	Cangyan mountain, Jingxing, Hebei	N 37°50′ E 114°07′	4	HeB1-1~HeB1-4
S6	Qingta, she, Hebei	N 36°47′ E 113°45′	4	HeB2-1~HeB2-4
S7	Culai mountain, Taian, Shandong	N 36°06′ E 117°16′	4	SD1-1~SD1-4
S8	Chishang, Zibo, Shandong	N 36°21′ E 118°04′	5	SD2-1~SD2-5
S9	Longquan, Yantai, Shandong	N 37°20′ E 121°47′	3	SD3-1~SD3-3
S10	Chengjiao, Tongbai, Henan	N 32°22′ E 113°23′	3	HeN1-1~HeN1-3
S11	Laowan, Tongbai, Henan	N 32°27′ E 113°19′	4	HeN2-1~HeN2-4
S12	Guxian, Tongbai, Henan	N 32°25′ E 113°37′	5	HeN3-1~HeN3~5
S13	Taohuatan, Jing, Anhui	N 30°31′ E 118°11′	4	AH1-1~AH1-4
S14	Langya mountain, Chuzhou, Anhui	N 32°16′ E 118°16′	5	AH2-1-AH2-5
S15	Jinzhai, Anhui	N 31°43′ E 115°55′	1	AH3-1
S16	Tongcheng, Anhui	N 31°02′ E 116°55′	5	AH4-1~AH4~5
S17	Zhang mountain, Hong’an, Hubei	N 31°14′ E 114°38′	4	HuB1~HuB4
S18	Anren, Hunan	N 26°42′ E 113°15′	2	HuN1, HuN2
S19	Tianpu, Wuning, Jiangxi	N 29°27′ E 115°11′	4	JX1~JX4
S20	Pingyang, Rongjiang, Guizhou	N 26°17′ E 108°20′	4	GZ1-1~GZ1~4
S21	Caotang, weng’an, Guizhou	N 27°10′ E 107°33′	2	GZ2-1, GZ2~2
S22	Lingui, Guangxi	N 25°14′ E 110°12′	3	GX1~GX3
S23	Liancheng, Guangnan, Yunnan	N 24°03′ E 105°01′	4	YN1~YN4

## Supplementary Materials

The following are available, Table S1: Contents (mg/g) of 8 types of saponins in 89 samples.
