# Supplementary material for: Quantitative and Chemical Fingerprint Analysis for the Quality Evaluation of Platycodi Radix Collected from Various Regions in China by HPLC Coupled with Chemometrics
Source: Molecules. 2018 Jul 23;23(7):1823. doi: 10.3390/molecules23071823 (PMC6099642; doi:10.3390/molecules23071823)
Supplement: Supplementary file 1 [file molecules-23-01823-s001.pdf]

Supplementary Table S1. Contents (mg/g) of 8 types of saponins in 89 samples.

Code	The contents of each compound[a]（mg/g）								Total
	1	2	3	4	5	6	7	8	
JL1-1	0.092	1.757	0.317	0.526	0.111	0.220	0.496	0.748	4.267
JL1-2	1.443	3.036	0.275	0.663	0.133	0.419	0.629	0.817	7.415
JL1-3	2.710	3.178	0.565	1.120	0.183	0.563	0.768	1.400	10.487
JL1-4	1.932	2.113	0.261	0.629	0.297	0.266	1.429	0.557	7.484
JL1-5	1.500	2.880	0.416	0.906	0.225	0.393	2.004	0.655	8.979
JL2-1	2.772	3.503	0.233	0.891	0.091	0.230	0.388	0.515	8.623
JL2-2	0.926	4.204	0.409	1.474	0.122	0.249	1.973	0.673	10.03
JL2-3	1.176	1.768	-[b]	0.282	-	0.166	0.582	0.485	4.459
JL2-4	0.695	2.454	0.230	0.327	-	0.204	0.396	0.600	4.906
JL2-5	0.385	1.850	0.211	1.096	-	0.173	0.440	0.746	4.901
JL2-6	0.916	2.220	-	0.466	-	0.155	0.438	0.360	4.555
JL3-1	1.058	2.701	0.165	0.577	-	0.162	0.413	0.385	5.461
JL3-2	0.317	2.316	0.206	0.551	-	0.200	0.545	0.719	4.854
JL3-3	1.546	3.165	0.297	0.537	-	0.227	0.527	0.628	6.927
JL3-4	1.987	3.141	0.359	0.589	0.145	0.712	1.073	0.597	8.603
JL4-1	1.949	3.082	0.223	0.450	0.132	0.215	0.788	0.541	7.380
JL4-2	1.369	3.102	0.192	0.460	0.093	0.352	0.708	0.688	6.964
JL4-3	1.457	3.984	0.171	0.673	-	0.187	0.764	0.833	8.069
JL4-4	0.928	2.261	0.161	0.732	-	0.184	0.595	0.429	5.290
HeB1-1	0.422	2.167	-	0.540	-	-	0.653	0.195	3.977
HeB1-2	0.827	3.975	0.126	0.731	0.106	0.138	1.199	0.343	7.445
HeB1-3	0.626	2.812	0.099	0.421	0.085	0.151	0.549	0.394	5.137
HeB1-4	0.630	2.619	0.109	0.445	0.134	0.148	0.943	0.268	5.296
HeB2-1	0.187	1.337	-	0.463	-	0.198	0.539	2.104	4.828
HeB2-2	0.320	1.218	0.134	0.400	-	-	0.234	0.274	2.580

HeB2-3	0.355	1.314	0.152	0.631	0.081	0.184	0.628	0.493	3.838
HeB2-4	0.763	1.623	0.200	0.841	0.103	0.210	0.987	0.500	5.227
SD1-1	0.430	2.085	0.330	0.979	-	0.235	0.688	0.636	5.383
SD1-2	0.175	1.526	-	1.085	-	0.120	0.505	0.307	3.718
SD1-3	0.207	1.196	0.339	1.013	0.102	0.601	1.616	1.194	6.268
SD1-4	0.379	1.535	0.072	0.788	-	0.071	0.474	0.664	3.983
SD2-1	0.252	1.905	0.081	0.410	-	0.207	0.738	0.630	4.223
SD2-2	0.832	1.995	0.121	0.404	0.248	0.082	1.656	0.661	5.999
SD2-3	1.834	2.104	0.188	0.665	0.444	-	1.561	0.418	7.214
SD2-4	0.583	2.438	-	0.318	-	0.171	0.975	0.756	5.241
SD2-5	0.633	2.027	0.090	0.394	-	0.184	0.499	0.610	4.437
SD3-1	0.530	1.420	0.115	0.637	0.271	0.238	3.157	1.065	7.433
SD3-2	0.717	1.482	0.161	0.593	0.304	0.217	2.660	0.714	6.848
SD3-3	0.544	1.844	0.111	0.589	0.275	0.246	3.100	0.856	7.565
HeN1-1	0.771	1.683	0.392	1.957	-	0.156	0.910	0.799	6.668
HeN1-2	0.327	3.172	0.210	0.928	0.117	0.267	3.563	0.645	9.229
HeN1-3	0.190	0.994	0.115	0.596	-	-	0.509	1.320	3.724
HeN2-1	0.112	0.823	-	0.378	-	0.138	0.794	0.454	2.699
HeN2-2	0.429	1.701	0.225	0.991	-	0.166	0.542	0.554	4.608
HeN2-3	0.577	1.707	0.498	0.943	-	0.171	0.674	0.658	5.228
HeN2-4	1.428	3.245	0.383	1.647	0.145	0.506	1.024	1.966	10.344
HeN3-1	1.356	2.559	0.952	1.699	0.545	0.263	2.900	1.585	11.859
HeN3-2	1.169	2.329	0.700	2.221	0.330	0.503	3.027	1.329	11.608
HeN3-3	1.250	2.895	0.576	1.158	0.373	0.140	1.645	0.880	8.917
HeN3-4	1.143	2.274	0.660	1.676	0.680	0.164	4.165	1.452	12.214
HeN3-5	0.704	1.854	0.311	1.685	0.209	0.306	1.925	1.081	8.075
AH1-1	0.309	1.123	0.187	0.868	-	0.209	0.783	0.739	4.218

AH1-2	0.336	1.690	0.211	1.353	0.097	0.302	0.838	0.851	5.678
AH1-3	0.747	1.532	0.291	0.597	0.220	0.214	1.200	0.757	5.558
AH1-4	0.248	1.277	0.108	0.523	-	0.155	0.691	0.520	3.522
AH2-1	0.494	2.093	0.144	0.588	-	-	0.645	0.540	4.504
AH2-2	0.392	3.031	0.102	1.285	-	0.179	1.033	0.818	6.840
AH2-3	0.410	0.419	0.104	1.013	0.153	0.301	1.715	1.051	5.166
AH2-4	0.462	1.937	0.119	1.157	0.129	0.183	1.242	0.734	5.963
AH2-5	0.651	2.727	0.157	0.731	-	0.120	0.691	0.707	5.784
AH3-1	0.375	1.916	1.158	1.918	-	0.133	1.252	1.609	8.361
AH4-1	0.586	3.595	0.088	1.497	0.165	0.871	1.476	1.953	10.231
AH4-2	1.996	2.194	0.980	1.967	0.210	0.464	1.275	1.990	11.076
AH4-3	1.975	3.565	0.428	2.265	0.166	0.775	1.395	2.171	12.740
AH4-4	0.159	0.579	3.355	5.650	1.263	1.355	5.156	2.122	19.639
AH4-5	0.901	2.920	0.141	1.107	0.129	0.460	0.870	1.143	7.671
HuB1	1.310	3.373	0.147	1.071	0.153	0.412	1.160	1.120	8.746
HuB2	2.689	4.064	0.179	1.138	0.073	0.313	0.781	1.166	10.403
HuB3	1.879	4.291	0.155	1.541	0.099	0.335	1.217	0.993	10.510
HuB4	0.585	2.119	0.143	0.738	0.086	0.213	0.656	1.041	5.581
HuN1	0.297	1.781	0.202	0.977	-	0.171	0.331	0.828	4.587
HuN2	0.328	2.563	0.215	1.879	-	0.182	0.486	1.311	6.964
JX1-1	0.408	1.830	0.201	1.014	0.392	0.354	3.883	0.709	8.791
JX1-2	0.298	2.621	0.201	2.108	0.258	0.327	3.586	0.913	10.312
JX1-3	0.269	1.494	0.220	1.106	0.678	0.398	6.822	1.505	12.492
JX1-4	0.209	1.585	0.207	1.062	0.342	0.259	3.155	0.839	7.658
GZ1-1	1.539	1.020	0.835	1.170	1.103	0.143	2.672	0.801	9.283
GZ1-2	2.364	2.153	0.630	1.053	0.799	0.165	2.621	1.011	10.796
GZ1-3	0.931	1.220	0.463	0.854	0.867	0.142	3.123	1.226	8.826

GZ1-4	0.599	0.797	0.335	0.677	1.280	0.211	4.648	1.310	9.857
GZ2-1	0.946	2.225	0.307	1.175	0.287	0.255	2.722	0.917	8.834
GZ2-2	0.198	0.512	0.065	0.348	0.431	0.192	4.624	0.660	7.030
GX1	0.357	0.658	0.347	0.713	1.331	0.196	4.070	0.773	8.445
GX2	0.854	1.080	0.506	1.105	1.477	0.366	5.688	1.054	12.130
GX3	0.208	0.335	0.385	0.662	1.623	0.245	5.582	1.190	10.230
YN1	0.679	2.227	0.829	1.996	0.418	0.392	2.604	0.825	9.970
YN2	1.207	4.146	0.508	1.656	0.408	0.378	2.284	0.926	11.513
YN3	0.292	1.365	0.261	1.528	0.265	0.402	3.011	0.822	7.946
YN4	1.062	2.884	0.770	2.622	0.506	0.500	4.013	0.799	13.156

[a] (1) Deapioplatycoside E; (2) Platycoside E; (3) Deapioplatycodin D3; (4) platycodin D3; (5) Deapioplatycodin D; (6) platycodin D2; (7) Platycodin D; (8) Polygalacin D.

[b] undetected.
